# On the Virilescence and Rejuvenescence of Animals: A Contribution to the Knowledge of the Regular Metamorphoses of Organic Bodies

**Published:** 1838-07

**Authors:** 


### Art. IV.

*Ueber Virilescenz und Rejuvenescenz thierischer Körper. Ein Beitrag zur Lehre von der regelwidrigen Metamorphose organischen Körper.* Von Dr. Carl Wilhelm Mehliss, praktischen Artze, &c. in Liebenwerda.—*Leipzig*, 1838.

*On the Virilescence and Rejuvenescence of Animals: a Contribution to the Knowledge of the regular Metamorphoses of Organic Bodies.* By Dr. Charles William Mehliss, of Liebenwerda.—*Leipzig*, 1838. 8vo. pp. 114.

In animal bodies there are three distinct periods or epochs,—viz. of formation, of growth, and of decay. The less perfect animals cease to exist as soon as they have attained their full growth, and become capable of propagating their species: the act which marks the perfection of their development is but the prelude to their destruction. In the higher animals, however, a longer or shorter period elapses after they are no longer capable of procreating their species; a period of gradual decay and progressive exhaustion of all the powers of life. This period is longer in man than in any other animal, and presents some phenomena which are well deserving of attention. Dr. Mehliss has chosen two of these as the subjects of his work: the assumption of the characters of the male by aged females, and the reappearance of the characteristics of youth in the aged of both sexes: to the former he gives the name of *Virilescence*, to the latter that of *Rejuvenescence*. He might likewise have added another new term, indicative of the appearance of some of the virile characters in the female, as he has noticed this circumstance in his book; and he might have named this condition *Feminescence,* from the same analogy on which the other terms are framed.

On the subject of Virilescence, the author quotes the authority of Hippocrates and Aristotle, and traces the origin of the fables of the middle ages concerning a "mutatio sexus" to the facts which they have related, and to the mythological sayings of the Grecian poets: yet such a change was not entirely without support from ascertained facts: old women had been seen with beards, and their voices had assumed the manly character; persons who had been educated as females had been seen to assume all the attributes of men; and others, who as women had remained barren in marriage, became the fathers of children. Such instances as these, which a careful enquiry would have robbed of all their value as facts, were strengthened by the opinion of Galen that the parts of generation are the same in both sexes, and vary only in position, the same parts being situated externally in men which are placed inwardly in females: by that of Aristotle, that a woman is merely an imperfect man; and by the scholastic dogma, that nature, under all circumstances, aims at perfection. Eusebius Nieremberg and Ulysses Aldrovandus had done their best to confirm these errors, by tales of exotic animals in which this conversion of sexes was a natural characteristic; and the doctrine was not entirely abandoned, even by anatomists, before the end of the sixteenth century. The seventeenth century, however, saw the error completely dispelled; and since then the whole subject of mutation of sex, instead of being a tissue of fables, has become an interesting branch of natural history.

*Virilescence.* Those appearances which our author includes under the general name of virilescence occur in females in whom one or more of the peculiar characteristics of their own sex have passed away, and consist in the assumption of some peculiar qualities of the male. The assumed characters differ in different cases, but agree in bearing a close resemblance to some part of the male frame, other than the organs of generation themselves. Virilescence, therefore, can take place only in those animals which continue to live some time after the power of generation has been lost, and in which the sexual differences are sufficiently strongly marked, not merely in the organs of generation themselves, but in other parts of the frame. Consequently, it cannot occur in vegetables; for the difference of sex is here only marked in the sexual organs themselves. Kob, indeed, has compared the regular metamorphoses of the organs of generation of plants into other parts, (as, for instance, of styles into stamens, and the occurrence of male blossoms on the female plants of the class Diœcia,) with the appearance of virilescence in animals; but these deviations from the normal condition are original, and do not admit of comparison with the animal phenomena, which distinctly require the complete development of the sexual character at a former period of life. This condition is fulfilled only in the more perfect animals,—in birds, in the mammalia, and in man. In insects, the sexual difference is sufficiently clearly marked in the external conformation of their bodies; but their life, closing with the process of generation, is too short to exhibit the phenomena of virilescence. In some of the Crustacea, in fish, and in amphibia, observation will probably yet detect the existence of these changes. In all animals, however, and under all circumstances, virilescence is extremely rare.

In birds, the chief distinction between the sexes, after the differences between the parts of generation, consists in the plumage, which is more developed, and possesses more lively and varied colours, in the male than in the female. The size of the body, the character of the voice, and the occurrence of spurs on the feet, establish other, but less striking, distinctive marks. In each of these particulars, the female bird may simulate the outward appearance of the male, and to such an extent that a careful observation shall scarcely distinguish the sexes. Our author collects abundant proof of this fact from undoubted authorities: of these, the greater part occurred in the domestic fowl and common pheasant; others in other species of pheasant, in the turkey, peacock, and common duck. The partridge, wood-pigeon, starling, bustard, chaffinch, and four or five other birds, have been observed to assume the same peculiarities. The change of plumage was the most remarkable, as well as by far the most frequent, phenomenon; the alteration of the voice was nearly as common; the growth of spurs, comb, &c., is reported in more than one case. These changes began after the bird had ceased to lay eggs, and became more marked as it grew older. In some instances the transformation was so complete, that not only was it difficult to make a distinction between the real and the assumed sex, but the difficulty was scarcely less for birds of the same species; especially as the deception was increased by the masculine behaviour of the transformed birds. In a few cases, eggs were detected in the ovary; and, in a large proportion of instances, the parts of generation were found partially or entirely wasted away.

In mammalia, the signs of virilescence are neither of so frequent occurrence nor so strongly marked as in birds; and hitherto they have been seen only in the stag and roe. The male of these animals, as is well known, is distinguished by horns, of which there is no trace in the female. These parts make their first appearance with the first complete development of the organs of generation; fall off annually, at the conclusion of one rutting season; and are again reproduced, in a more perfect form, at the beginning of the next. Virilescence consists chiefly in the growth of these parts in the female: it shows itself, however, occasionally in other parts; for instance, in the hair. More than one of these changes have been seen in the same animal. In some instances both horns were produced; in others, only one, and that usually on the right side. Two instances only are adduced in which the phenomena of virilescence showed themselves in the hair of the animal. In one case, the hair of the head, neck, and abdomen, the shape of the ears and extremities, and the odour of the animal, gave it the closest possible resemblance to the male, and it followed the other females as if urged by sexual desire. Valmont de Bomare has described an animal which had one horn on the left side, and organs of generation closely resembling those of the male; the ovaries hung down like testicles, the clitoris was elongated, and the vagina contracted. In three instances, the animals were in calf. The case quoted from Valmont de Bomare is the only one in which the parts of generation underwent those changes which take place so frequently in birds. The statement that horns have made their appearance on the heads of animals which do not usually bear them,—as, for instance, the hare,—is justly treated as a fable; and the growth of horny excrescences on the skin of men and animals is shown to have no connexion with the phenomena of virilescence. The change of the colour of the skin, too, which is observed to take place in old animals of both sexes, is shown not to be allied to virilescence.

In the human species, the phenomena of virilescence consist in the growth of hair, partly on the face, in the form of a beard, and partly in other situations where, in ordinary circumstances, it does not exist to any extent, even in men; and in a strengthening of the tone of the voice, so as to resemble that of the male. The facts which are quoted by our author are not very numerous, amounting only to eight detailed cases and a few references to others. With the exception of the case of extirpation of the ovaries, related by Pott, the phenomena of virilescence are referrible to suppression of the menstrual discharge, occurring as well at an early period of life as in more advanced age. The growth of hairs on the upper lip in young persons, a short time before the appearance of the menstrual discharge, is regarded by the author as an event of not unfrequent occurrence.

The following general conclusions are drawn by Mehliss from the facts adduced by him:

1. The changes which take place in animal bodies during virilescence are either organic or dynamic. To the latter belong the changes of the voice and of the sexual characters. The former are of two kinds: in some cases a degeneration of structure occurs, as the gradual wasting away of the sexual organs; in other instances there is an actual development of parts which, in the natural state, exist only in males. The new growths which thus take place, either already existed in a rudimentary form, and are merely more fully developed, (for example, the comb in the common fowl, hair in women, crista pectoralis in the turkey;) or they already existed in the female under a different form, and merely undergo a change, (for instance, the feathers of birds;) or, lastly, they were originally absent, and must be considered as entirely new formations, (as horns in the hind, spurs in the common fowl.)

2. The changes take place gradually, obey the laws to which the development of the same parts in the male is subject, and follow the same order.

3. In all the individuals which have exhibited the phenomena of virilescence, the characters of their own sex were already fully developed; the greater number, too, had been fruitful; and it is only in some individuals of the human species that the power of reproduction has been wanting.

4. The appearance of virilescence was, in all cases, attended either by an entire loss, or a remarkable diminution of the power of reproduction; and, as the virilescence increased, the power of reproduction diminished, at length entirely disappeared, and never returned.

5. On the other hand, virilescence seems to have commenced, in most instances, before the usual period of the disappearance of the sexual functions; so that it did not begin with the commencement of *old* age but a longer or shorter time before it. In men, and in the mammalia, it occurs proportionably earlier than in birds.

6. All the individuals in whom virilescence has been observed have been placed under favorable circumstances. They all appear to have been remarkably robust, powerful, and healthy, and to have reached a good old age.

7. In no case was the health of the individual impaired by virilescence; nor was the occurrence of the phenomena accompanied by feelings or symptoms of sickness.

*Feminescence.* After closing his account of Virilescence, the author notices more briefly the reverse condition, or the assumption of some of the male characters by the female, which we might term *Feminescence.* The two principal circumstances noticed under this head are, the appearance of the catamenia, or, at least, of a sanguineous discharge in the male; and the secretion of milk by the male breast. Our author quotes the authority of the ancients for the occurrence of the first-named phenomenon; but not a single credible instance, as might be believed, is adduced. The most curious statement under this head is the assertion by certain authors that the catamenial lustration from the penis was inflicted on the *Jews* as a divine punishment! On the other hand, the secretion of milk in the breast of males is a fact sufficiently attested, both by ancient and modern writers. Here, also, credulity has added her legends. In the sixteenth century, some missionaries in Brazil asserted that there was a whole Indian nation whose women had small and withered breasts, and whose children owed their nourishment entirely to the males! It is, however, true that one of the best authenticated instances of this fact occurred in a South American, and is related by Humboldt. Francisco Lozano, aged thirty-two, a peasant of a small village in Cumana, nourished his child with his own milk. His wife, immediately after her delivery, fell sick; and Lozano, in the hope of quieting the child, applied it to his breast. A secretion of milk took plaçe, which gradually increased until it afforded sufficient nourishment for the infant. Humboldt and Bonpland were assured by eye-witnesses that during five months the child took no other nourishment whatever. Humboldt saw both the father and son; and states that the breasts of the former closely resembled those of a female. We may add the following very similar fact, on the authority of one of our most enterprising travellers. A young Chipewyan lost his wife in her first pregnancy: he applied the infant to his breast, to still its cries; and "the force of the powerful passion by which he was actuated, produced the same effect in his case as it has done in some others which are recorded: a flow of milk actually took place from his breast." "Our informant, Mr. Wenzel, added that he had often seen this Indian in his old age, and that his left breast even then retained the unusual size it had acquired in his occupation of nurse.*" Some remarkable examples also are given of male animals who had given suck either to the young of their own or of other species, or had furnished milk to man. The occurrence of this abnormal secretion was in no case accompanied by a change in the functions or structure of the parts of generation.

We cannot follow our author in his examination of the somewhat analogous, but really very different subject of *Hermaphrodism:* neither can

* Franklin's Voyages, vol. ii. p. 54.—*Richardson's Journal.*

we give his discussion on the causes of the singular change to Virilescence. We give the conclusion to which he arrives, in his own words, and we hope it may be more satisfactory to the reader than it is to us. "Virilescence," says Dr. Mehliss, "like many other degenerations to which organic bodies are liable, is a means of compensating a disproportion which exists in the organism between the energy of the vegetative life of the whole body and that of individual organs."

*Rejuvenescence.* The idea of the assumption by aged persons of the characters of youth was familiar to the ancients, and probably formed the groundwork of the fable of Medea and Æson. Pliny and his successors have related instances of the kind, and modern writers have added to our stock of facts. No one, however, we believe, before Dr. Mehliss, has attempted to collect the scattered cases to ascertain their credibility, and thus to make the whole subject a branch of physiology. He arranges the phenomena of rejuvenescence under five distinct heads:—1. The secretion of milk by aged females. 2. The return of the menstrual discharge. 3. The cutting of teeth in old age. 4. The growth of hair similar in colour to that of the young. 5. The sharpening of the intellect, and restoration of the vivacity of youth to the old.

It would occupy too much space were we to notice, however briefly, the various facts which are here detailed; and we must refer our readers to the original work. The subjoined table shows, at one view, the number and nature of the principal facts.

| | Number of instances of | | | |
|---|---|---|---|---|
| | Dentition of the aged. | | Lactation by aged females. | Menstruation of aged females. |
| Age of the individuals. | Males. | Females. | | |
| Between 40 and 50 | 0 | 4 | | |
| — 50 ... 60 | 1 | 4 | 2 | 1 |
| — 60 ... 70 | 3 | 2 | 1 | 0 |
| — 70 ... 80 | 3 | 2 | 0 | 7 |
| — 80 ... 90 | 6 | 2 | 0 | 0 |
| — 90 ... 100 | 1 | 1 | 0 | 1 |
| Above 100 | 6 | 1 | 0 | 1 |
| Total | 20 | 16 | 3 | 10 |

It is stated by Dr. Mehliss to be a necessary condition for the appearance of the phenomena of rejuvenescence, that there exists complete energy and integrity of vegetative life at the period of decrepitude. It is also shown that the mode in which this energy shows itself depends upon local causes. The secretion of milk, the return of the menses, and the growth of the teeth, may in most cases be attributed to an irritation applied to the parts concerned. In some instances, these changes are accompanied by symptoms of constitutional irritation, and fatal results have followed in more than one case; a fact which connects the physiology of the subject with practical medicine.

Dr. Mehliss's book is, on the whole, curious and interesting; and contains some important physiological views: but it is disfigured with much that is fanciful, and fails not to exhibit abundant examples of the great defects of German literature—prosy tediousness of detail, endless repetitions, and vast wordiness.

